# Cationic Covalent Organic Framework as Fluorescent Sensor for the Detection of Poly- and Perfluoroalkyl Substances (PFAS)

**DOI:** 10.1063/4.0001128

**Published:** 2025-10-27

**Authors:** Maryam Piroozzadeh

**Affiliations:** university of texas at dallas, richardson, texas, USA

## Abstract

Abstract

Sensitive detection methods for “PFAS” are critical for evaluating the safety level of our water sources. LC-MS-MS is the method currently we are using for the detection of PFAS, but it requires highly trained laboratory personnel, expensive and complex instrumentation, difficult sample preparation, and tedious data collection. Therefore, alternative detection method based on fluorescent sensing have intrinsic advantages like fast response time, easy operation, cost effectiveness, naked eye identification, and the potential to build hand-held sensor devices for onsite detection. We have designed a novel cationic covalent organic framework (c-COF) based on perylene diimide The hydrophobic functionality and cationic charge assisted for the detection of both light weight PFCAs and PFOS.

